# Effects of aspirin on dementia and cognitive function in diabetic patients: the ASCEND trial

**DOI:** 10.1093/eurheartj/ehac179

**Published:** 2022-04-08

**Authors:** Sarah Parish, Marion Mafham, Alison Offer, Jill Barton, Karl Wallendszus, William Stevens, Georgina Buck, Richard Haynes, Rory Collins, Louise Bowman, Jane Armitage

**Affiliations:** MRC Population Health Research Unit, Nuffield Department of Population Health, University of Oxford, Big Data Institute, Old Road Campus, Roosevelt Drive, Oxford OX3 7LF, UK; Clinical Trial Service Unit and Epidemiological Studies Unit, Nuffield Department of Population Health, University of Oxford, Oxford, UK; Clinical Trial Service Unit and Epidemiological Studies Unit, Nuffield Department of Population Health, University of Oxford, Oxford, UK; Clinical Trial Service Unit and Epidemiological Studies Unit, Nuffield Department of Population Health, University of Oxford, Oxford, UK; Clinical Trial Service Unit and Epidemiological Studies Unit, Nuffield Department of Population Health, University of Oxford, Oxford, UK; Clinical Trial Service Unit and Epidemiological Studies Unit, Nuffield Department of Population Health, University of Oxford, Oxford, UK; Clinical Trial Service Unit and Epidemiological Studies Unit, Nuffield Department of Population Health, University of Oxford, Oxford, UK; Clinical Trial Service Unit and Epidemiological Studies Unit, Nuffield Department of Population Health, University of Oxford, Oxford, UK; Clinical Trial Service Unit and Epidemiological Studies Unit, Nuffield Department of Population Health, University of Oxford, Oxford, UK; Clinical Trial Service Unit and Epidemiological Studies Unit, Nuffield Department of Population Health, University of Oxford, Oxford, UK; MRC Population Health Research Unit, Nuffield Department of Population Health, University of Oxford, Big Data Institute, Old Road Campus, Roosevelt Drive, Oxford OX3 7LF, UK; Clinical Trial Service Unit and Epidemiological Studies Unit, Nuffield Department of Population Health, University of Oxford, Oxford, UK; MRC Population Health Research Unit, Nuffield Department of Population Health, University of Oxford, Big Data Institute, Old Road Campus, Roosevelt Drive, Oxford OX3 7LF, UK; Clinical Trial Service Unit and Epidemiological Studies Unit, Nuffield Department of Population Health, University of Oxford, Oxford, UK

**Keywords:** Aspirin, Dementia, Cardiovascular disease

## Abstract

**Aims:**

Aspirin is widely used in cardiovascular disease prevention but is also associated with an increased risk of bleeding. The net effect of aspirin on dementia and cognitive impairment is uncertain.

**Methods and results:**

In the ASCEND trial, 15 480 people from the UK with diabetes and no history of cardiovascular disease were randomized to aspirin 100 mg daily or matching placebo for a mean of 7.4 years. The 15 427 ASCEND participants with no recorded dementia prior to baseline were included in this cognitive study with a primary pre-specified outcome of ‘broad dementia’, comprising dementia, cognitive impairment, or confusion. This was ascertained through participant, carer, or general practitioner report or hospital admission diagnosis, by 31 March 2019 (∼2 years beyond the scheduled treatment period). The broad dementia outcome occurred in a similar percentage of participants in the aspirin group and placebo group: 548 participants (7.1%) vs. 598 (7.8%), rate ratio 0.91 [95% confidence interval (CI), 0.81–1.02]. Thus, the CI excluded proportional hazards of >2% and proportional benefits of >19%.

**Conclusion:**

Aspirin does not have a large proportional effect on the risk of dementia. Trials or meta-analyses with larger total numbers of incident dementia cases to increase statistical power are needed to assess whether any modest proportional 10–15% benefits of 5–7 years of aspirin use on dementia exist.

**Clinical Trial Registration:**

Current Controlled Trials number, ISRCTN60635500; ClinicalTrials.gov number: NCT00135226.

## Structured Graphical Abstract

**Structured Graphical Abstract:**
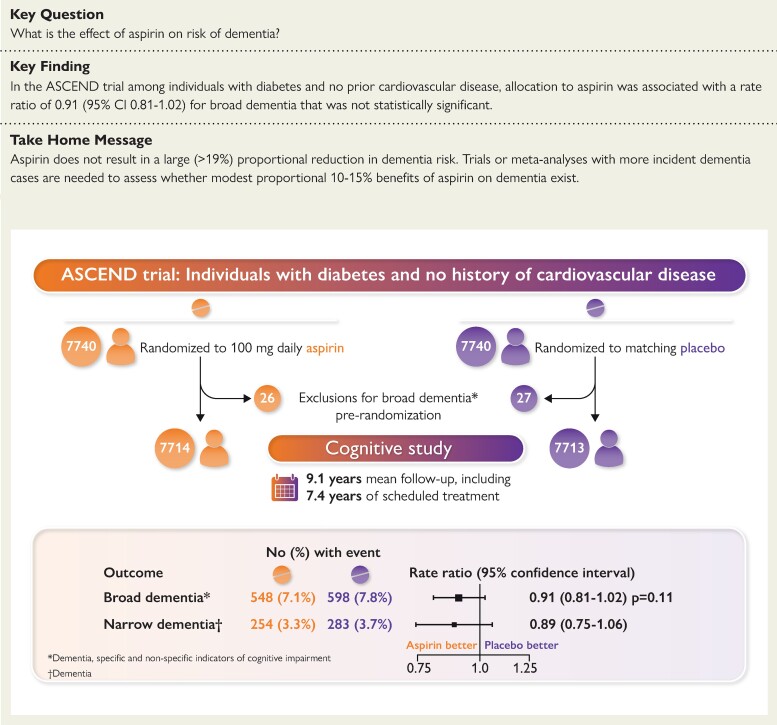
The effect of aspirin on dementia in the ASCEND trial: Study design and summary results.

**See the editorial comment for this article ‘The end of aspirin for dementia prevention in diabetes?’, by Steen D. Kristensen *et al*., https://doi.org/10.1093/eurheartj/ehac211.**

## Introduction

In primary cardiovascular disease prevention, daily low-dose aspirin has been shown, in a recent meta-analysis of randomized trials, to cause an 11% proportional decrease in the risk of major vascular events (including a 19% reduction in ischaemic strokes) but a 43% proportional increase in the risk of serious bleeding.^1^ Cerebrovascular events are associated with cognitive impairment, and thus, aspirin may prevent cognitive impairment through the avoidance of ischaemic strokes, transient ischaemic attacks (TIAs), and microinfarcts, but could cause cognitive impairment through an increased risk of intracranial haemorrhage and cerebral microbleeds.^2–6^ Despite being in widespread use for cardiovascular prevention, it is not known whether aspirin has a net benefit or hazard on dementia and cognitive function. Previous randomized trials of aspirin have not convincingly detected any effect of aspirin use on dementia incidence or cognitive impairment, but this has only been assessed in studies with fewer than 600 dementia cases.^7–9^

Diabetes increases the rate of cognitive decline and risk of dementia and so any effect of aspirin on dementia may be more marked and important among people with diabetes.^10,11^ This report, among the 15 427 participants with diabetes but without prior reported dementia or cognitive impairment who had been randomized in the ASCEND (A Study of Cardiovascular Events in Diabetes) trial, assesses the effect of randomization to daily low-dose aspirin for an average of 7.4 years on risk of dementia and other indicators of cognitive impairment. Additionally, we report the observational associations of different types of non-fatal vascular and bleeding events occurring during the trial with subsequent dementia risk.

## Methods

The ASCEND randomized trial assessed the effect of daily low-dose aspirin (and, in a 2 × 2 factorial design, omega-3 fatty acid supplementation) on the risk of serious vascular events in people with diabetes who did not have known atherosclerotic cardiovascular disease.^12,13^ The protocol was approved by the North West Multi-centre Research Ethics Committee. All of the participants provided written informed consent. This report is for the pre-specified ASCEND cognitive study,^14^ which includes additional outcome data for dementia and cognitive impairment identified from linked electronic health records and results of cognitive function tests assessed among surviving participants being actively followed up at the end of the scheduled treatment period.

### Participants and procedures

CONSORT guidelines for reporting have been followed (see Supplementary material online, *Table S1* and Methods S1).^12,13,15^ Full details of the study procedures have been reported previously.^12,15^ Briefly, men and women who were at least 40 years of age were considered eligible for ASCEND if they had received a diagnosis of any type of diabetes mellitus but did not have known cardiovascular disease. Following a mail-based recruitment and screening process and an 8- to 10-week placebo run-in, participants who remained willing and eligible and were adherent to the run-in medication were randomized to 100 mg of aspirin or matching placebo tablet once daily (and, in a 2 × 2 factorial design, omega-3 fatty acid supplementation or placebo). Participants then received 6-monthly mailings of their allocated tablets and capsules and a follow-up questionnaire, until the end of the scheduled treatment period. The questionnaires sought information regarding adherence to the study treatments, all hospitalizations and serious adverse events, and any symptomatic bleeding episodes for which the participants sought medical assistance. Where participants (or their carers) were unable to complete the questionnaire, follow-up was via questionnaire mailed to their general practitioner (GP). Additional electronic hospital episode (eHE) coded data for death, cancer, and hospitalizations were obtained from NHS Digital, the NHS Wales Informatics Service, and Public Health Scotland. For this study, eHE data were available for a mean of 14 years before randomization, throughout the study treatment period (until final follow-ups between January and July 2017 after a mean of 7.4 years) and for a further ∼2 years until 31 March 2019. The additional 2-year follow-up was available in 99.5% of surviving participants, after censoring 44 from Northern Ireland and 22 who withdrew consent for long-term follow-up at the end of the study treatment period.^14^ This additional 2 years of data was to help capture dementia cases arising during the scheduled treatment period but not recorded in a hospital admission until 1 or 2 years later, since a previous study indicated a mean delay of 1.6 years between the first mention of dementia in hospital admission records compared with primary care records.

#### Cognitive testing at the end of the scheduled treatment period

Efforts were made to obtain a cognitive assessment in all survivors at the time of the final follow-up where this was being undertaken by the participant (or their carer), but this was not possible for participants whose information was being provided by their GP. Participants who provided a valid e-mail address were invited to complete the online ‘Healthy Minds’ cognitive function test developed by UK Biobank.^16^ Participants who did not complete an online assessment were telephoned by a study nurse who administered the Telephone Interview of Cognitive Status (TICSm) questionnaire^17^ and the verbal fluency (VF) test.^18^

The Healthy Minds online cognitive function test is a recently developed battery of cognitive function tests that has been used to assess 100 000 healthy adults enrolled in the UK Biobank study.^16^ The battery used for ASCEND included five tests: (i) fluid intelligence; (ii) trail making; (iii) symbol-digit substitution; (iv) pairs matching; and (v) numeric memory.^19^ The TICSm is a 13-item test covering 4 component domains: orientation, memory (registration, recent memory, and delayed recall), attention/calculation, and language (semantic memory, comprehension, and repetition).^17^ In the VF test, participants are asked to name in 1 min as many different animals as they can and the score is the number named.

#### Dementia and cognitive impairment outcomes

**Figure 1:**
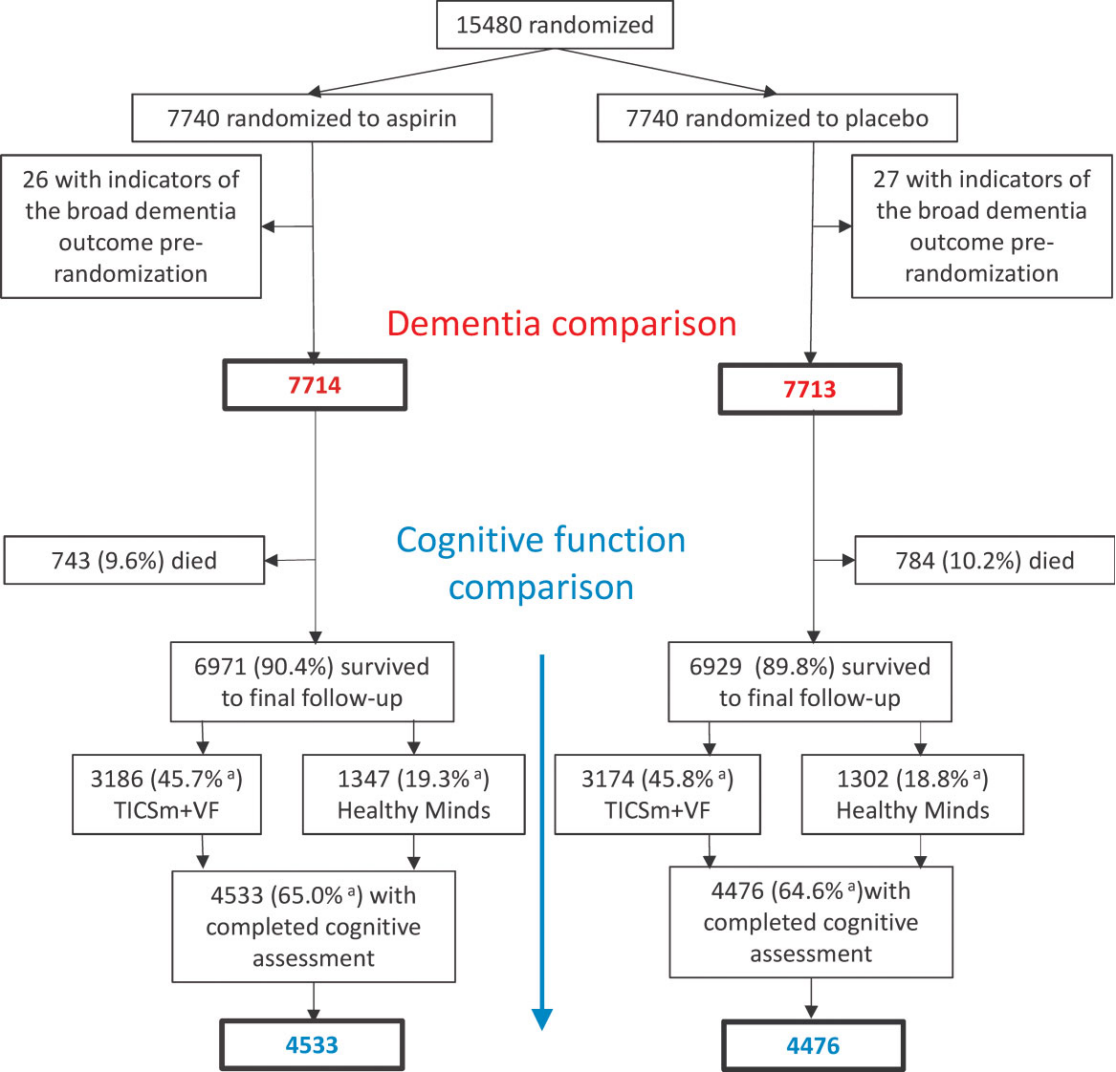
Study profile. ^a^Per cent of those not known to have died.

In the ASCEND Cognitive Data Analysis Plan,^14^ the primary dementia outcome encompassed dementia, and specific and non-specific indicators of cognitive impairment, referred to as ‘broad dementia’. It was defined as the first occurrence post-randomization of dementia, cognitive impairment, or delirium/confusion recorded on a follow-up questionnaire; or taking dementia medication (reported at a mean of 6.5 years post-randomization); or referral to a memory clinic (asked specifically on the final follow-up questionnaire); or cognitive impairment cited as a reason for not completing the cognitive assessment; or an ICD-10 diagnosis of dementia (Alzheimer’s, vascular, and unspecified) or a non-specific indication of cognitive impairment (delirium or senile degeneration of brain/unspecified degenerative disease of nervous system) in eHE data or recorded on the death certificate (codes shown in Supplementary material online, *Table S2*); or any hospital episode indicating discharge to geriatric psychiatry. The 53 (26 allocated aspirin, 27 allocated placebo) participants randomized into ASCEND with a record meeting this broad dementia outcome prior to randomization were excluded from this report (*Figure 1*).

The secondary outcome was a cognitive *z*-score among participants who attempted cognitive testing (see Supplementary material online, Methods S2). Additional defined outcomes were a narrower dementia outcome excluding delirium/confusion and non-specific indications of cognitive impairment (see Supplementary material online, *Table S2*) and an outcome comprising broad dementia or discharge to care home. Diagnoses of dementia-related outcomes from eHE data or from death certificates were pre-specified to be included up to 31 March 2019.^14^ The study was estimated to have 80% power to detect proportional 15% higher or 18% lower risks of dementia in the aspirin compared with placebo groups at 2*P* < 0.05 (see Supplementary material online, Methods S3).

### Statistical analysis

#### Randomized comparisons

The log-rank method was used to conduct intention-to-treat comparisons in time-to-event analyses of the first occurrence of each dementia outcome among participants in the aspirin and placebo arms.^20,21^

The components of the TICSm and VF tests (TICSm + VF) were combined into a cognitive function *z*-score, following a previously published method,^22^ and likewise the Healthy Minds test components were combined into a *z*-score (see Supplementary material online, *Table S3* and Methods S2). The effects of aspirin allocation on each of the cognitive *z*-scores were estimated using a linear regression with adjustment for single years of age at cognitive test and sex and the results for the two types of cognitive test combined in an inverse-variance fixed-effects meta-analysis.

#### Observational analyses

In observational analyses of the associations of non-fatal serious vascular events, arterial revascularization procedures, and major bleeds during the scheduled treatment period with subsequent first reports of dementia outcomes, follow-up was censored before the first occurrence of a disabling stroke or disabling intracranial bleed (see Supplementary material online, Methods S1) because, in such participants, the disability is already recognized.

As dementia diagnoses that might otherwise not have been recorded until sometime later, or never recorded, may be recorded incidentally during hospital admissions triggered by other reasons (such as cardiovascular events or bleed events) and this could be a potential bias, a method of analysis was devised to control for the number of these admissions. The main analyses were adjusted for the number of hospital admissions not listing a dementia-related diagnosis in the primary position. A sensitivity analysis adjusted for the number of hospital admissions for any cause. Dementia-related diagnoses during the same hospital episode as the non-fatal event under consideration were ignored (on the basis that diagnosis was brought forward by having the non-fatal event). Observational analyses were undertaken of the association of various non-fatal events of interest (non-haemorrhagic stroke, TIA, myocardial infarction, coronary and non-coronary revascularization, and intracranial, gastrointestinal, and other major bleeds) with subsequently recorded dementia. The analyses used the Poisson regression, using 2-year periods of age-at-risk spent with or without the event, and were adjusted for: indicators of the prior occurrence of each of the other non-fatal events of interest post-randomization (0 = no prior event, 1 = prior event, and for events during the interval, values between 0 and 1 for the fraction of the interval after the event); the number of hospital admissions during the interval (0, 1, ≥2; see Supplementary material online, Methods S4); randomized treatment allocation; sex; prior diseases; and baseline predictors of dementia including a hospital diagnosis-based frailty score^23^ (see Supplementary material online, Methods S4 and *Table S4*). Sensitivity analyses examined associations within strata by the number of hospital admissions during an interval.

In the primary report from ASCEND, daily low-dose aspirin was associated with a 12% lower proportional and 1.7% lower absolute risk of a serious vascular event or revascularization and a 29% higher proportional and 0.9% higher absolute risk of major bleeds.^12^ To estimate the impact on the risk of broad dementia associated with differences in non-fatal event occurrence, the estimated rate ratio (RR) for the broad dementia outcome associated with the occurrence of each of the types of event was applied to the observed absolute differences in the incidence of non-fatal serious vascular events, revascularizations, and major bleeds between aspirin and placebo-assigned participants.

## Results

Among the 15 427 participants in these analyses, the mean age was 63 [standard deviation (SD) 9] years and 37% were women, with no differences between those allocated aspirin vs. placebo (*Table 1*). The mean adherence to aspirin or placebo during the 7.4 years of scheduled follow-up was 70% in each group.^12^ Follow-up by the trial procedures was 99.1% complete and 99.7% were linked to eHE data providing additional follow-up.

**Table 1:** Completeness of follow-up for dementia and cognitive testing by allocated treatment and occurrence of intracranial events

	Aspirin	Placebo	Total	Total without disabling intracranial event^a^
**All participants** ^b^	7714	7713	15 427	15 357
Age at randomization, mean (SD), years	63.2 (9.2)	63.3 (9.2)	63.2 (9.2)	63.2 (9.2)
Female sex, *n* (%)	2883 (37.4)	2894 (37.5)	5777 (37.4)	5752 (37.5)
With linkage to electronic hospital episodes, *n* (%)	7691 (99.7)	7689 (99.7)	15 380 (99.7)	15 310 (99.7)
In-trial hospitalizations per linked individual, median (IQR)^c^	3 (1–6)	3 (1–6)	3 (1–6)	3 (1–6)
Any record of hospitalization in trial, *n* (%)	6284 (81)	6265 (81)	12 549 (81)	12 479 (81)
Disabling intracranial event^a^ in trial, *n* (%)	27 (0.4)	43 (0.6)	70 (0.5)	0 (0.0)
Non-disabling intracranial event^d^ in trial, *n* (%)	340 (4.4)	378 (4.9)	718 (4.7)	718 (4.7)
Died during follow-up, *n* (%)	743 (9.6)	784 (10.2)	1527 (9.9)	1497 (9.7)
Surviving to final follow-up (FFU), *n* (%)	6971 (90.4)	6929 (89.8)	13 900 (90.1)	13 860 (90.3)
**Participants surviving to FFU**	6971	6929	13 900	13 860
FFU from participant/carer, *n* (%)	5750 (82.5)	5670 (81.8)	11 420 (82.2)	11 404 (82.3)
Undertaking Healthy Minds cognitive testing, *n* (%)	1347 (19.3)	1302 (18.8)	2649 (19.1)	2647 (19.1)
Undertaking TICSm + VF cognitive testing, *n* (%)	3186 (45.7)	3174 (45.8)	6360 (45.8)	6355 (45.9)
Undertaking any cognitive testing, *n* (%)	4533 (65.0)	4476 (64.6)	9009 (64.8)	9002 (64.9)
Age at cognitive testing, mean (SD), years	69.6 (8.1)	69.4 (8.2)	69.5 (8.1)	69.5 (8.1)
Education post-16, *n* (%)^e^	2826 (62.3)	2812 (62.8)	5638 (62.6)	5643 (62.6)
Disabling intracranial event^a^ in trial, *n* (%)	17 (0.2)	23 (0.3)	40 (0.3)	0 (0.0)

a Disabling stroke or subdural haemorrhage.

b Of the 15 480 participants randomized in ASCEND, 26 participants from the aspirin arm and 27 from the placebo arm had the broad dementia outcome in the eHE data prior to randomization and have been excluded from all the present analyses.

c Association between at least one post-randomization admission to hospital and randomized allocation to aspirin, rate ratio = 0.99 (95% CI, 0.96–1.03).

d Non-disabling stroke, transient ischaemic attack, or subdural haemorrhage.

e Only available in those with cognitive testing, percentage is of the number of individuals undertaking cognitive testing.

During the mean of 9.1 (interquartile range 8.0–10.3) years of follow-up (i.e. 7.4 years in-trial and 1.7 years post-trial), the broad dementia outcome was recorded from trial sources in 463 participants and from the eHE data in 960 participants, with ∼60% of the former (277 participants) recorded in both sources (see Supplementary material online, *Table S5*). The mean age at the first report of dementia was 76 (SD 9) years. About 81% of participants in each treatment group had a hospitalization during follow-up, providing similar opportunities for incidental diagnosis of dementia outcomes in the two treatment groups (*Table 1*).

### Randomized effects of aspirin on dementia outcomes

**Figure 2:**
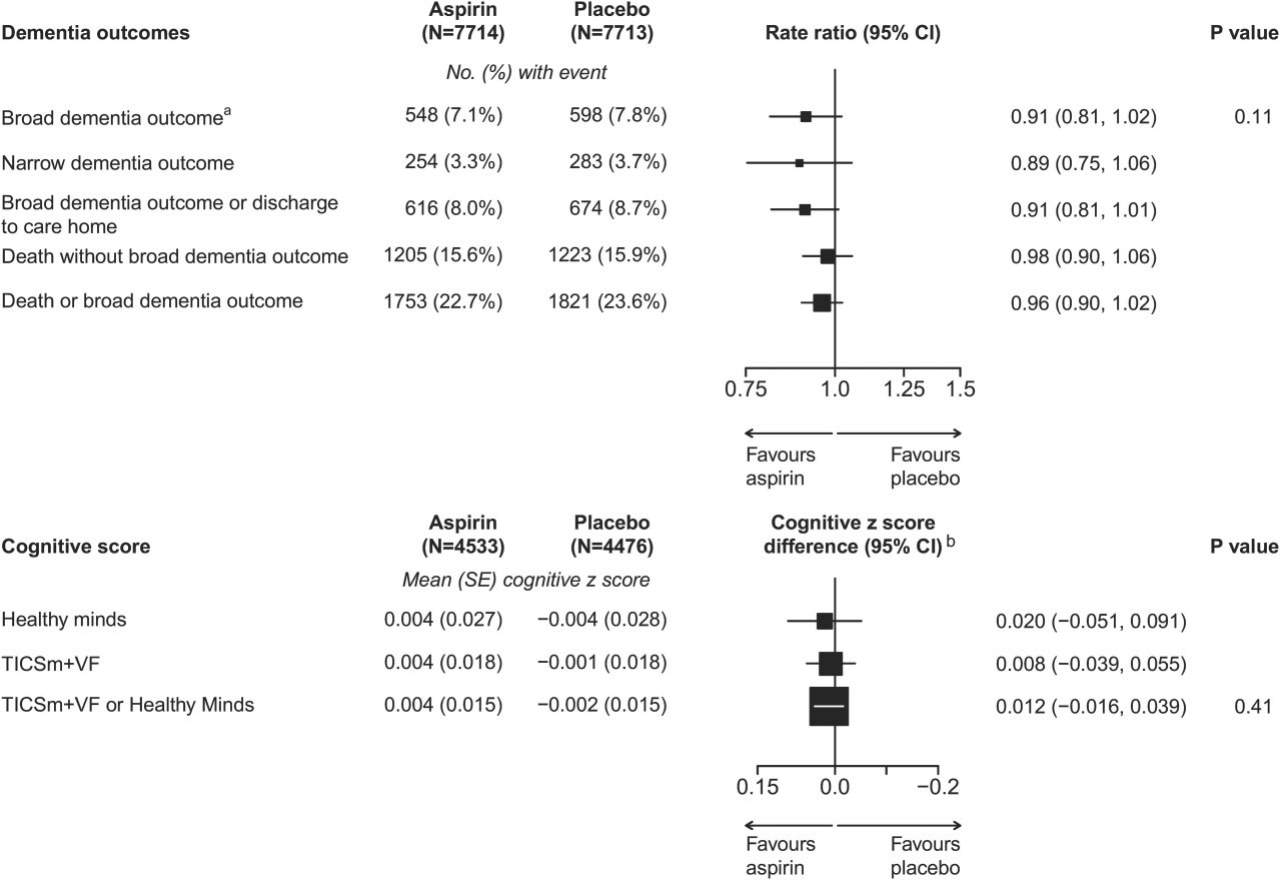
Effect of randomization to aspirin on dementia and cognitive function. Box sizes for the rate ratios are proportional to the amount of statistical information. ^a^Fifty-three individuals (26 on aspirin, 27 on placebo) are censored due to the broad dementia outcome before randomization. ^b^Cognitive *z*-score difference is adjusted for age at test and sex. The axis reversed to maintain the direction of ‘favours aspirin’ to the left.

The broad dementia outcome occurred in 548 (7.1%) participants in the aspirin group and in 598 (7.8%) in the placebo group [RR, 0.91, 95% confidence interval (CI) 0.81–1.02; *P* = 0.11, *Figure 2*]. The results were similar in comparisons of the more restricted, narrower dementia outcome [254 (3.3%) vs. 283 (3.7%), RR 0.89 (95% CI 0.75–1.06)], and the combination of broad dementia and discharge to care home [616 (8.0%) vs. 674 (8.7%), RR 0.91 (0.81–1.01)]. The percentage of participants dying without a prior (or concomitant) recording of dementia was similar in the aspirin and placebo groups (15.6% vs. 15.9%). The combined outcome of broad dementia or death occurred in 1753 (22.7%) participants in the aspirin group and in 1821 (23.6%) in the placebo group (RR, 0.96, 95% CI 0.90–1.02).

### Completeness of cognitive function at final follow-up and randomized effects of aspirin

Among the 90% of participants surviving to final follow-up, 65% of participants undertook a cognitive assessment (19% Healthy Minds and 46% TICSm + VF; *Table 1* and Supplementary material online, *Table S6*).

The end of study (at a mean of 7.8 years from randomization) cognitive function *z*-score difference between the aspirin and placebo groups was 0.012 (95% CI −0.016 to 0.039, *P* = 0.41) in the combined analysis of both types of the test [deriving from differences of 0.020 (95% CI −0.051 to 0.091) in those undertaking Healthy Minds, and 0.008 (95% CI −0.039 to 0.055) in those undertaking TICSm + VF; *Figure 2*].

Among participants without broad dementia recorded prior to cognitive testing, there was a log-linear relationship between lower TICSm + VF cognitive *z*-score and higher rate of the subsequent first recording of the broad dementia (see Supplementary material online, *Figure S1*). Participants completing the Healthy Minds test had a lower risk of dementia. In contrast, participants not completing cognitive testing had a high risk of dementia.

### Observational associations of in-trial incident events with subsequent dementia and cognitive ageing

**Figure 3:**
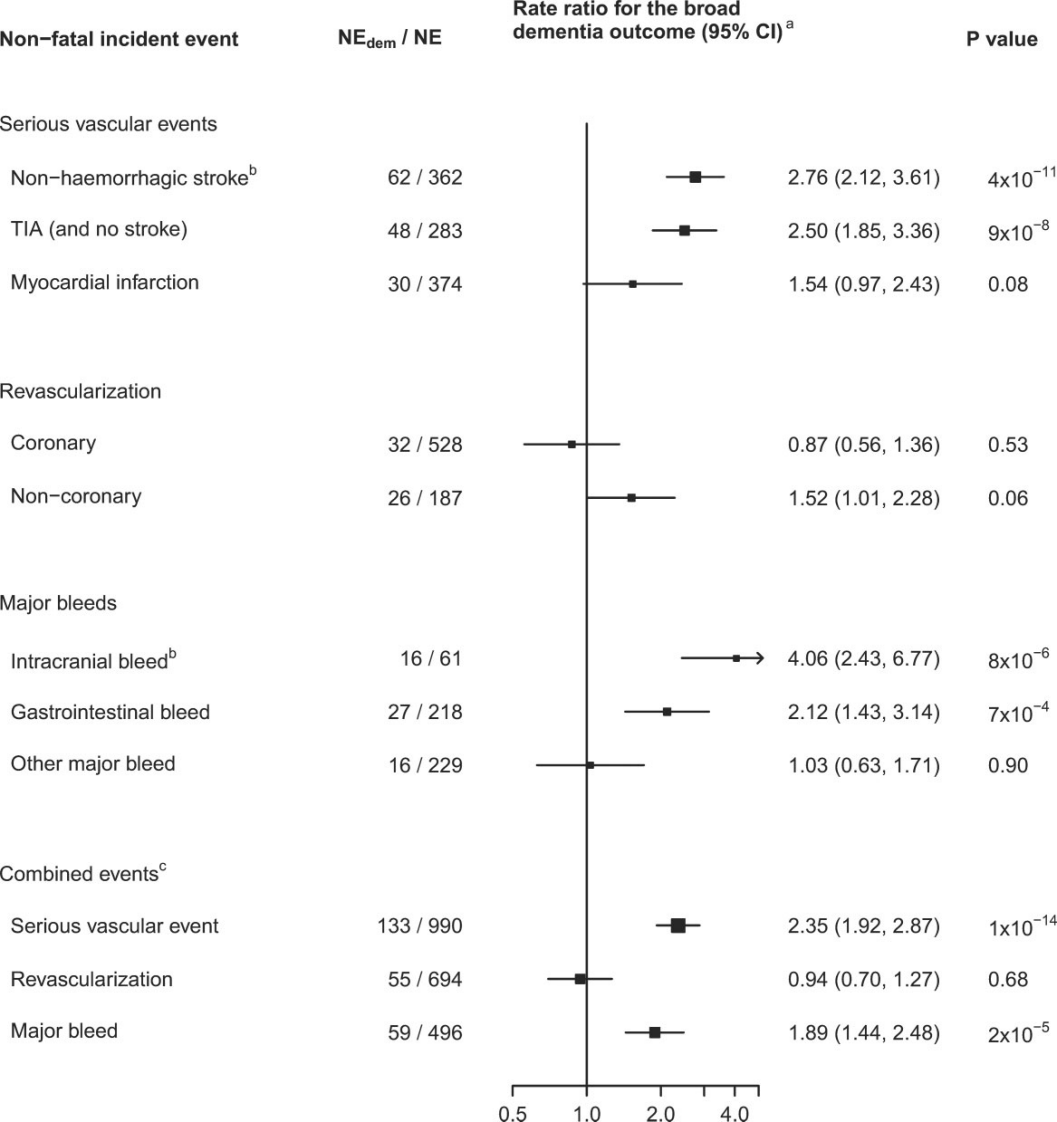
Association of the incidence of vascular and bleed events with dementia. NE_dem_, number of participants with the non-fatal incident event and, subsequently, the broad dementia outcome; NE, total number of patients with the non-fatal incident event. ^a^Adjusted for age, sex, randomized allocation, number of hospitalizations, incidence of the other non-fatal incident events, prior disease, and baseline predictors of dementia selected by backwards selection with *p*_sel_ = 0.2. ^b^Non-disabling events (analyses are censored at disabling stroke or intracranial bleed). Intracranial bleed includes haemorrhagic stroke. ^c^Rate ratios associated with having the combined events tend to be higher than those with individual types because, for people with more than one type of event, they encompass the risks associated with each of the types of event.

Among participants with no disabling intracranial event during in-trial follow-up (*Table 1*), all of the incident events considered, except coronary revascularization and ‘other’ (not intracranial or gastrointestinal) major bleed, were associated with higher RRs for the broad dementia outcome compared with those not suffering that event (*Figure 3*), after adjustment for the baseline predictors of dementia shown in Supplementary material online, *Table S4*. The highest RRs for the broad dementia outcome were observed for non-fatal non-haemorrhagic stroke [RR 2.76 (95% CI, 2.12–3.61), *P* = 4 × 10^−11^, *n* = 362] and non-fatal intracranial bleed [RR 4.06 (2.43–6.77), *P* = 8 × 10^−6^, *n* = 61]. Having a non-fatal TIA (but no stroke, *n* = 283), a myocardial infarction (*n* = 374), a non-coronary revascularization (*n* = 187), or a gastrointestinal bleed (*n* = 218) were each associated with RRs of between 1.5 and 2.5 for the broad dementia outcome. The combined events of serious vascular event (*n* = 990), any arterial revascularization (*n* = 694), and major bleed (*n* = 496) were, respectively, associated with RRs of 2.35 (95% CI, 1.92–2.87), 0.94 (0.70–1.27), and 1.89 (1.44–2.48) (*Figure 3*).

Sensitivity analyses showed that the RRs were similar (i) with different levels of adjustment for baseline predictors of dementia (see Supplementary material online, *Table S7*) and (ii) within strata by frequency of hospital admissions for other reasons and remained similar when adjusted for the number of all hospital admissions (see Supplementary material online, *Figure S2*). The RRs associated with the non-fatal events were similar when discharge to care home was included in the outcome and for the narrow dementia outcome (see Supplementary material online, *Figure S3*).

### Estimated association of events avoided and caused by aspirin with dementia

The percentages of participants in the ASCEND cognitive study avoiding non-fatal serious vascular events, avoiding revascularizations, or suffering a major bleed through assignment to aspirin therapy were 0.57, 0.55, and 0.91%, respectively (see Supplementary material online, *Table S8*). If the relationships in *Figure 3* reflect causal, reversible associations, then the net effect of these observed event differences with aspirin would be estimated to result in an RR for broad dementia of 1.001 (0.998–1.004) (see Supplementary material online, *Table S8*).

## Discussion

Allocation to daily low-dose aspirin for a mean of 7.4 years had no statistically significant effect (at 2*P* < 0.05) on the risk of the broad or narrow dementia outcome, or of the combination of the broad dementia outcome and discharge to care home. The RR of 0.91 (95% CI, 0.81–1.02) observed for the broad dementia outcome makes a net proportional harm >2% or a net proportional benefit >19% unlikely (*Structured Graphical Abstract*). However, the wide CI means that potentially clinically important benefits of up to 18% proportional reductions in risk of dementia from 5 to 7 years of aspirin therapy are still possible. Nor was there any statistically significant difference in cognitive function at the end of the trial between the aspirin and placebo arm {mean cognitive function *z*-scores [0.004 (SE 0.015) vs. −0.002 (0.015)], difference 0.012 (95% CI, −0.016 to 0.039)}.

**Figure 4:**
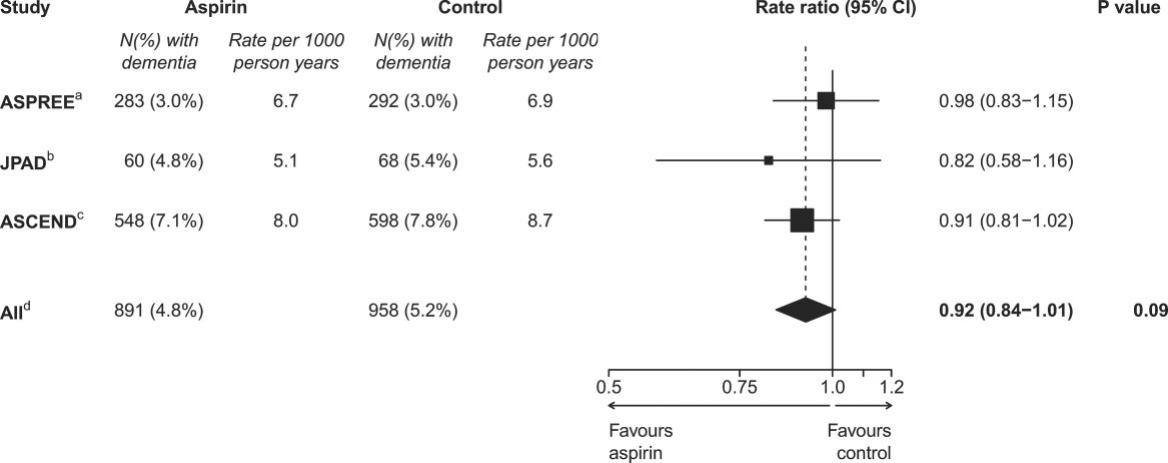
Effect of randomization to aspirin on dementia in a meta-analysis of three trials. Box sizes for the rate ratios are proportional to the amount of statistical information. ^a^ASPREE trial^7^: a randomized placebo-controlled trial of 100 mg daily aspirin in 19 114 healthy elderly individuals with a median follow-up of 4.7 years. The secondary endpoint of dementia is used. ^b^JPAD trial^8^: a randomized open-label trial of 80–100 mg daily aspirin in 2536 patients with diabetes with a median follow-up of 11.4 years. ^c^The ASCEND broad dementia outcome is used. ^d^Fixed-effects meta-analysis of ASPREE, JPAD, and ASCEND.

These ASCEND results represent one of the most comprehensive randomized assessments to date of the effects of aspirin for cardiovascular disease prevention on cognitive outcomes. Among the 12 other trials included in a recent meta-analysis of the effects of low-dose aspirin on the primary prevention of cardiovascular disease,^1^ only two have reported on dementia, each finding no statistically significant effect of aspirin: the ASPREE (Aspirin in Reducing Events in the Elderly) trial^7^ found a hazard ratio of 0.98 (95% CI 0.83–1.15) based on 575 cases over ∼5 years of trial follow-up and the open-label Japanese Primary Prevention of Atherosclerosis with Aspirin for Diabetes trial^8^ found a hazard ratio of 0.82 (95% CI 0.58–1.16), based on 128 dementia cases over ∼14 years of trial and post-trial follow-up. Meta-analysis of these results with those for broad dementia in ASCEND yielded a combined hazard ratio of 0.92 (95% CI 0.84–1.01) (*P* = 0.09) (*Figure 4*).

Three of the 12 trials in the meta-analysis of the effects of aspirin on the primary prevention of cardiovascular disease included a cognitive assessment, which was in 17 701 participants (with at least one follow-up cognitive assessment) in the ASPREE trial,^24^ in 5226 in the Women’s Health Study,^25^ and in 2262 in the Aspirin for Asymptomatic Atherosclerosis^26^ trial, and all found no statistically significant effect of aspirin on cognition. A meta-analysis in 2017 of the effects of aspirin on cognitive function in a broader range of randomized trials also found no evidence for any effect.^9^

In observational analyses within the trial population, having a serious vascular event or a major bleed were each associated with about a two-fold increase in proportional risks of the dementia outcomes. This suggests that the estimated benefit on dementia risk from the effects of aspirin treatment in reducing the risk of serious vascular events and revascularizations might be somewhat counterbalanced by its effect from increasing the risk of major bleeds. However, these observational analyses do not take account of any effects of aspirin mediated via subclinical events such as unreported TIAs, silent brain infarcts, microinfarcts, microbleeds, or cumulative small vessel disease.^5,27,28^

Linkage to routinely collected electronic hospital admission data provided coverage for the capture of dementia by this route for 99.7% of participants. However, dementia diagnoses recorded from hospital admissions may be delayed, meaning that some dementia events recorded in the study could have occurred prior to randomization. Furthermore, ∼20% of dementia cases may be missed when hospital admissions data alone are used to ascertain dementia cases.^29,30^ Linkage to primary care would have provided a more complete and timely capture of dementia but was not available with national coverage at the time of this study. As the number of hospital admissions was similar in the aspirin and placebo arm, this should not have biased the randomized comparison, and the observational analyses mitigated this issue by adjusting for the frequency of hospital admissions.

The cognitive function comparison in the study was limited by only having a simple cognitive function assessment at the end of the scheduled treatment period and therefore could not use change in cognitive function as an outcome. As discussed in the context of three other cardiovascular trials,^22^ more extensive cognitive testing on multiple occasions would be likely to lead to a somewhat more sensitive cognitive comparison. ASCEND was a low-cost mail-based study, and a further limitation was that cognitive assessment conducted only at the end of the study was achieved in only 58% of the study population because 10% of participants had not survived and additional more costly tactics to pursue non-respondents were not feasible. Among 586 survivors who had suffered a non-disabling intracranial event in the trial (but no disabling event), only 52% completed cognitive testing (data not shown) compared with 65% in all survivors, indicating some response bias in the cognitive assessment, and furthermore, non-responders had a higher than the average rate of recorded dementia (see Supplementary material online, *Figure S1*). For the assessment of cognitive outcomes in large low-cost trials, acquiring dementia incidence from routinely collected healthcare data may be more worthwhile than undertaking cognitive testing.

## Conclusions

Allocation to aspirin for a mean of 7.4 years was not associated with any statistically significant differences in the risk of dementia and the results excluded net proportional benefits of 19% or more. This is consistent with findings in the small number of other trials that have investigated the effects of aspirin on dementia. Trials or meta-analyses of trials with larger numbers of incident dementia cases would provide greater statistical power to assess whether any modest proportional 10–15% benefits of 5–7 years of aspirin use on dementia exist, which might translate into greater cumulative absolute benefits of longer-term use.

## Writing Committee (on behalf of the ASCEND Study Collaborative Group)

S. Parish^†^, M. Mafham^†^, A. Offer, J. Barton, K. Wallendszus, W. Stevens, G. Buck, R. Haynes, R. Collins^‡^, L. Bowman^‡^, and J. Armitage^‡^ (^†^equal first author, ^‡^equal senior author). S. Parish had full access to all the data in the study and takes responsibility for its integrity and the data analysis.

## Supplementary material

Supplementary material is available at *European Heart Journal* online.

## Supplementary Material

ehac179_Supplementary_Data

## Data Availability

Proposals for data access will be considered by the ASCEND Steering Committee in accordance with the trial protocol. Procedures for accessing the data are available at: https://www.ndph.ox.ac.uk/files/about/ndph-data-access-policy-1.pdf.
