# Supplementary material for: Effects of aspirin on dementia and cognitive function in diabetic patients: the ASCEND trial
Source: Eur Heart J. 2022 Apr 8;43(21):2010–9. doi: 10.1093/eurheartj/ehac179 (PMC9242621; doi:10.1093/eurheartj/ehac179)
Supplement: ehac179_Supplementary_Data [file ehac179_supplementary_data.pdf]

Supplement

Contents

Methods S1. Further details on study procedures

Methods S2. Cognitive tests and z scores

Methods S3. Prior power calculation of the randomized comparison

Methods S4. Further details of statistical analysis

Supplementary References

Table S1. Consort checklist

Table S2. Definitions of the dementia outcomes

Table S3. Test metrics and binary missing indicator flags in the Healthy Minds assessment

Table S4. Baseline predictors of the broad dementia outcome selected by different criteria

Table S5. Sources of dementia and cognitive impairment

Table S6. Numbers attempting and mean time taken for the individual components of the Healthy Minds cognitive assessment among the 2649 individuals included in the Healthy Minds analysis.

Table S7. Sensitivity analysis showing the observational association of the incidence of vascular and bleed events with dementia with different levels of adjustment for the predictors of dementia.

Table S8. Estimated benefit for the broad dementia outcome of the effects of low dose aspirin on events in the ASCEND study

Figure S1. Association of Healthy Minds and TICSm+VF cognitive function z scores at end of study with the first subsequent recording of the broad dementia outcome.

Figure S2. Observational association of the incidence of vascular and bleed events with dementia within strata by the number of hospital admissions for other causes during two-year age-at-risk intervals.

Figure S3. Observational association of the incidence of vascular and bleed events with various dementia outcomes.

Methods S1. Further details on study procedures

The final protocol for the ASCEND trial was approved prior to the unblinding of treatment allocation and was published with the main reports, which also included details of protocol amendments during the trial.[1, 2] The Data Analysis Plan for the trial was also published prior to unblinding.[3] Funding to add a cognitive study was obtained during the trial and a separate Data Analysis Plan for the ASCEND Cognitive Study was agreed and posted online in August 2018 prior to the publication of the ASCEND main reports.[4] The plan overlooked specification that participants with broad dementia prior to randomization should be excluded from the cognitive analysis but this revision was implemented prior to the unblinding in May 2021 of analyses of the defined cognitive outcomes (which involved additional electronic health record data received up to April 2021).

Patients were randomized centrally at the CTSU, University of Oxford, using a minimization algorithm to ensure balance by prognostic variables as described previously.[3] Individuals were randomized between June 24 2005 and July 28 2011 and followed up until 2017. Participants, care providers and those assessing outcomes were blind to the random allocations to treatment or placebo.

Individuals were assessed for cognitive function at the end of the trial either by a web-based assessment (6360 individuals assessed between 28 March 2017 and 21 December 2017), or by the modified telephone interview for cognitive status[3] (2649 individuals assessed between 28 March 2017 and 27 December 2017). Dementia was identified from follow-up forms during the trial, and death certificates and linkage to electronic hospital episode statistics until 31 March 2019.

In the ASCEND trial strokes and intra-cranial bleeds were classified as non-disabling where there seemed to be no help needed with everyday activities (including where no information was available).[1]

Methods S2. Cognitive tests and z scores

Cognitive tests

The online cognitive function test ("Healthy Minds") is a recently developed battery of cognitive function tests that has been used to assess 100,000 healthy adults enrolled in the UK Biobank study.[5] All tests were constructed using established testing paradigms that have been shown to produce valid scores and to be acceptable to participants.[6] The Healthy Minds online cognitive function assessment used for ASCEND includes 5 tests given in the order: (i) fluid intelligence; (ii) trail making; (iii) symbol-digit substitution; (iv) pairs matching; and (v) numeric memory.[7] Participants may abandon an individual test (which might indicate they found it difficult) and proceed to the next test during the assessment. Hence, potentially informative partial data will be available for some participants. (In the UK Biobank study an additional reaction time test has been found to be unreliable and so this test was not included in ASCEND.)

Cognitive function z scores

The TICS-m is a 13-item test covering four component domains: orientation, memory (registration, recent memory and delayed recall), attention/calculation and language (semantic memory, comprehension and repetition); test score ranges are from 0-39 with a higher score indicating better cognitive function. In the VF test participants are

asked to name in one minute as many animals as they can and the score is the number of different animals named.

From the 5 Healthy Minds tests, 13 metrics and 5 binary flags to indicate completion of the tests are used (Table S3). As abandoning a test could be an indication of having difficulty, and hence be informative, the approach tries to include partial information as far as possible. Participants are regarded as having attempted the testing if they complete at least one test or provide usable partial data on at least two tests. For the fluid intelligence test, the test was considered partial but usable if some questions had been answered before the test was abandoned. Similarly for the numerical memory and symbol digit substitution, the tests were considered partial but usable if some levels had been completed before the test was abandoned. For trail making, if the test was abandoned mid-trial but a time was recorded for a partial trail, the time taken was divided by the fraction of the trail that had been completed. For participants who attempted testing, a principal component analysis was conducted over the 18 items of data from the tests, as a data reduction technique. The first principal component (signed so that a higher value indicates better cognitive function) can be regarded as a measure of general cognitive function and is taken as the score.[6] (This has mean zero and standard deviation of one and so is a z score).

For the TICS-m based score separate z scores for TICS-m and VF were computed by subtracting their mean and dividing by their standard deviation and forming a weighted average of the two z scores with weights 4:1 to reflect the 4 domains covered by TICS-m.

Methods S3. Prior power calculation of the randomized comparison

We assumed that the proportion of patients with broad dementia outcome by the end of the study would be 6.5% in the placebo group (based on information available on the blinded event rate by August 2018) and determined that a sample size of 15 500 patients would provide the trial with a power of 80% at a two-tailed P value of less than 0.05 to detect an 18% lower or 15% higher risk of dementia in the aspirin group than in the placebo group.[4]

Methods S4. Further details of statistical analysis

Baseline factors associated with dementia function

Baseline factors associated with the broad dementia outcome were identified to allow adjustment for them in the observational analyses. These factors were identified using backwards selection and Poisson regression as for the main analyses (with adjustment for the number of hospital admissions for other reasons), with age at entry (as single years), sex, diabetes type, duration and management, and prior diseases forced into the model and with the cut-off for removal of the other variables being a p-value of 0.2. In addition to the factors forced into the model, 79 additional variables were tested (Table S4). Stricter selection criteria, using backwards selection with a p-value 0.001 for removal and stepwise selection with a p-value of 0.001 for selection/removal, were also investigated (p-values of approximately 0.05/number of variables available for selection; Table S4[8]).

Supplementary References

1. ASCEND Study Collaborative Group. Effects of Aspirin for Primary Prevention in Persons with Diabetes Mellitus. *N Engl J Med*. 2018; **379**: 1529-39.

2. ASCEND Study Collaborative Group. Effects of n-3 Fatty Acid Supplements in Diabetes Mellitus. *N Engl J Med*. 2018; **379**: 1540-50.
3. ASCEND Study Collaborative Group. ASCEND: A Study of Cardiovascular Events iN Diabetes: Characteristics of a randomized trial of aspirin and of omega-3 fatty acid supplementation in 15,480 people with diabetes. *Am Heart J*. 2018; **198**: 135-44.
4. ASCEND Study Collaborative Group. ASCEND Cognitive Data Analysis Plan. 2018 24 August 2018 [cited 27 January 2022]; Available from: https://ascend.medsci.ox.ac.uk/professionals
5. UK Biobank study. [cited 27 January 2022]; Available from: http://www.ukbiobank.ac.uk/
6. Lyall DM, Cullen B, Allerhand M, Smith DJ, Mackay D, Evans J, et al. Cognitive Test Scores in UK Biobank: Data Reduction in 480,416 Participants and Longitudinal Stability in 20,346 Participants. *PLoS One*. 2016; **11**: e0154222.
7. UK Biobank - Cognitive function online. [cited 27 January 2022]; Available from: http://biobank.ctsu.ox.ac.uk/crystal/label.cgi?id=116
8. Gilbert T, Neuburger J, Kraindler J, Keeble E, Smith P, Ariti C, et al. Development and validation of a Hospital Frailty Risk Score focusing on older people in acute care settings using electronic hospital records: an observational study. *Lancet*. 2018; **391**: 1775-82.

Table S1: CONSORT 2010 checklist of information to include when reporting a randomized trial*

Section/Topic	Item No	Checklist item	Reported in section:
**Title and abstract**			
	1a	Identification as a randomized trial in the title	Title
	1b	Structured summary of trial design, methods, results, and conclusions	Abstract
**Introduction**			
Background and objectives	2a	Scientific background and explanation of rationale	Introduction
	2b	Specific objectives or hypotheses	Last paragraph of introduction
**Methods**			
Trial design	3a	Description of trial design (such as parallel, factorial) including allocation ratio	1st paragraph of methods
	3b	Important changes to methods after trial commencement (such as eligibility criteria), with reasons	None are very relevant to this cognitive study. Methods S1 notes that they are detailed in the main ASCEND report (ref 12).
Participants	4a	Eligibility criteria for participants	Methods - Participants and Procedures
	4b	Settings and locations where the data were collected	Participants and procedures
Interventions	5	The interventions for each group with sufficient details to allow replication, including how and when they were actually administered	Methods - Participants and Procedures
Outcomes	6a	Completely defined pre-specified primary and secondary outcome measures, including how and when they were assessed	Methods – Participants and Procedures - *Dementia and cognitive outcomes*, Table S2-S3, Methods S3
	6b	Any changes to trial outcomes after the trial commenced, with reasons	Methods S1
Sample size	7a	How sample size was determined	Methods S3
	7b	When applicable, explanation of any interim analyses and stopping guidelines	NA
Randomization:			
Sequence generation	8a	Method used to generate the random allocation sequence	Methods S1
	8b	Type of randomization; details of any restriction (such as blocking and block size)	
Allocation	9	Mechanism used to implement the random allocation sequence (such as sequentially numbered containers),	Methods S1

concealment mechanism		describing any steps taken to conceal the sequence until interventions were assigned	
Implementation	10	Who generated the random allocation sequence, who enrolled participants, and who assigned participants to interventions	Methods S1
Blinding	11a	If done, who was blinded after assignment to interventions (for example, participants, care providers, those assessing outcomes) and how	Methods S1
	11b	If relevant, description of the similarity of interventions	NA
Statistical methods	12a	Statistical methods used to compare groups for primary and secondary outcomes	Methods- Statistical analysis, Methods S4
	12b	Methods for additional analyses, such as subgroup analyses and adjusted analyses	Methods - Observational analyses, Methods S4
**Results**			
Participant flow (a diagram is strongly recommended)	13a	For each group, the numbers of participants who were randomly assigned, received intended treatment, and were analysed for the primary outcome	Figure 1
	13b	For each group, losses and exclusions after randomization, together with reasons	Figure 1
Recruitment	14a	Dates defining the periods of recruitment and follow-up	Methods, Methods S1
	14b	Why the trial ended or was stopped	It reached its target as specified in its protocol
Baseline data	15	A table showing baseline demographic and clinical characteristics for each group	Table 1, prior publications
Numbers analysed	16	For each group, number of participants (denominator) included in each analysis and whether the analysis was by original assigned groups	Figure 1
Outcomes and estimation	17a	For each primary and secondary outcome, results for each group, and the estimated effect size and its precision (such as 95% confidence interval)	Figure 2
	17b	For binary outcomes, presentation of both absolute and relative effect sizes is recommended	Figure 2
Ancillary analyses	18	Results of any other analyses performed, including subgroup analyses and adjusted analyses, distinguishing pre-specified from exploratory	Figure 3, Table S8
Harms	19	All important harms or unintended effects in each group (for specific guidance see CONSORT for harms)	Included in cited main ASCEND reports (refs 12-13)
**Discussion**			
Limitations	20	Trial limitations, addressing sources of potential bias, imprecision, and, if relevant, multiplicity of analyses	Discussion
Generalisability	21	Generalisability (external validity, applicability) of the trial findings	Discussion includes a meta-analysis of three trials

Interpretation	22	Interpretation consistent with results, balancing benefits and harms, and considering other relevant evidence	Discussion
**Other information**			
Registration	23	Registration number and name of trial registry	Abstract
Protocol	24	Where the full trial protocol can be accessed, if available	Methods S1 notes that it is published in ref 12
Funding	25	Sources of funding and other support (such as supply of drugs), role of funders	Acknowledgements and funding

* CONSORT 2010 checklist, see www.consort-statement.org.

Table S2. Definitions of the dementia outcomes

		Broad dementia outcome (primary outcome)	Narrow dementia outcome
**Electronic Hospital episode data[a] and death certificate ICD10 codes**			
	Alzheimer's disease		
F00	Dementia in Alzheimer's disease	Y	Y
F000A	Dementia in Alzheimer's disease with early onset	Y	Y
F001A	Dementia in Alzheimer's disease with late onset	Y	Y
F002A	Dementia in Alzheimer's disease, atypical or mixed type	Y	Y
F009A	Dementia in Alzheimer's disease, unspecified	Y	Y
G30	Alzheimer's disease	Y	Y
G30.0	Alzheimer's disease with early onset	Y	Y
G30.1	Alzheimer's disease with late onset	Y	Y
G30.8	Other Alzheimer disease, unspecified	Y	Y
G30.9	Alzheimer's disease unspecified	Y	Y
	Vascular dementia		
F01	Vascular dementia	Y	Y
F010	Vascular dementia of acute onset	Y	Y
F011	Multi-infarct dementia	Y	Y
F012	Subcortical vascular dementia	Y	Y
F013	Mixed cortical and subcortical vascular dementia	Y	Y
F018	Other vascular dementia	Y	Y
F019	Vascular dementia, unspecified	Y	Y
I67.3	Binswanger's disease	Y	Y
	Unspecified dementia type		
F03X	Unspecified dementia	Y	Y
F051	Delirium superimposed on dementia	Y	Y
	Non-specific indicators of cognitive impairment		
F050	Delirium not superimposed on dementia, so described	Y	
F058	Other delirium	Y	
F059	Delirium unspecified	Y	
G31.1	Senile degeneration of brain, not otherwise classified	Y	
G31.9	Degenerative disease of nervous system, unspecified	Y	

Table S2 (continued)

	Broad dementia outcome (primary outcome)	Narrow dementia outcome
**Electronic hospital episode data[a] discharge information**		
Discharge to geriatric psychiatry	Y	
Discharge to care home[b]		
**ASCEND trial outcomes**		
Dementia or cognitive impairment recorded on any follow-up form	Y	Y
Confusion or disorientation recorded on any follow-up form	Y	
Donepezil hydrochloride recorded on follow-up form	Y	Y
Referral to memory clinic recorded on final follow-up form	Y	
Cognitive impairment cited as a reason for not completing cognitive assessment	Y	

Abbreviations: SAE: serious adverse event

[a] Includes data from the Hospital Episode Statistics (HES) from England and Patient Episode Database for Wales (PEDW). [b] Discharge to care home contributes to the broad dementia plus care home outcome only

Table S3. Test metrics and binary missing indicator flags in the Healthy Minds assessment

Test metric[a]	Partial information used [b]
Fluid intelligence score	Y
Fluid intelligence number attempted	Y
Trail making time for round 1: inverse	Y
Trail making time for round 2: inverse	Y
Trail making score round 1: negative of number of errors	Y
Trail making score round 2: negative of number of errors	Y
Symbol-digit substitution number correct	Y
Symbol-digit substitution attempts made	Y
Pairs matching score for round 1: negative of number of errors	
Pairs matching score for round 2: negative of number of errors	
Pairs matching completed round 3[c]: binary flag	
Pairs matching score for round 3: negative of number of errors	
Numeric memory score (maximum number of digits remembered)	Y
**Binary missing indicator flags for all data**	
Fluid intelligence score complete	
Trail making complete	
Symbol-digit substitution complete	
Pairs matching complete	
Numeric memory complete	

[a] These metrics were selected as the main results from the information returned by the Healthy Minds procedure and, where indicated, were transformed so that higher values corresponded to a greater quantity of successful test attained. [b] Y=included even when participant abandoned mid test.. [c] Pairs round 3 is offered only to participants making 0 or 1 errors on round 2, hence indicator is regarded as a data item.

Table S4: Baseline predictors of the broad dementia outcome selected by different criteria, for use as adjustment in the observational analyses

	Main adjustment	Alternative adjustments	
**Predictor**	**Backwards selection p_{sel}=0.2**	**Backwards selection p_{sel}=0.001**	**Stepwise selection p_{sel}=0.001**
Body mass index	Y		
Weight	Y		
Quintiles of weight	Y	Y	
Townsend deprivation index (grouped)	Y	Y	Y
Prior alcohol misuse	Y		
Smoking (current / former / never)	Y		Y
Quintiles of systolic blood-pressure	Y		
Imputed blood-pressure	Y	Y	Y
Imputed diabetes duration	Y		
Hospital admission frailty index (grouped)	Y	Y	Y
Vascular risk group	Y		
Prior Liver disease	Y		Y
Prior lower GI bleed	Y		
Total cholesterol	Y		
Non-HDL cholesterol	Y		
apoB:apoA$_1$ ratio	Y		
HbA1c (grouped)	Y		
Estimated glomerular filtration rate (eGFR)	Y		
eGFR (grouped)	Y		
ACE/ARB use at baseline	Y	Y	
Gastroprotective use at baseline	Y	Y	
NSAID use at baseline	Y		
Proton pump inhibitor use at baseline	Y		
Statin use at baseline	Y		
Sulphonylurea use at baseline	Y		

Abbreviations: p_{sel} : the p value for selection (and removal in stepwise selection); ACE: Angiotensin-converting enzyme inhibitors; ARB:Angiotensin II Receptor Blockers; NSAID: Non-steroidal anti-inflammatory drugs; apoB: apolipoprotein B; apoA$_1$: apolipoprotein A$_1$

In addition to the factors listed here, factors forced into the model were age at entry, sex, diabetes at entry (duration in years, type (1 or 2), and management), diabetic retinopathy, treated hypertension, and the following prior conditions from electronic hospital episode data: heart failure, atrial fibrillation, subdural haemorrhage and other major bleed. The analysis was also adjusted for the number of hospitalisations.

Variables available for selection in the backwards and stepwise selections included prior alcohol misuse, body mass index, weight and height, smoking, blood pressure variables, education (available for individuals who took the cognitive test only), Townsend deprivation index, prior diseases, estimated 5 year risk of serious vascular events, baseline medications, albumin, albumin:creatinine ratio, apoA$_1$, apoB, apoB:apoA$_1$ ratio, cholesterol (total, HDL and non-HDL) measurements, HbA1c, estimated glomerular filtration rate, grouped hospital admission frailty score[8]. Continuous variables were included both as values and grouped variables. Missing values were imputed with a missing flag as missingness can be informative. A total of 79 variables were tested.

Table S5. Sources of dementia and cognitive impairment

	Electronic hospital episode data categorization				
	**Broad dementia outcome**	**Narrow dementia outcome**	**Broad dementia outcome or carehome**	**No electronic hospital episode linkage**	**All**
Number of participants	960	468	1108	47	15427
Narrow dementia outcome on FU, No. (%)	177 (18.4%)	150 (32.1%)	180 (16.2%)	0 (0.0%)	219 (1.4%)
Broad dementia outcome on FU, No. (%)	277 (28.9%)	197 (42.1%)	285 (25.7%)	0 (0.0%)	463 (3.0%)
Died during trial, No. (%)	232 (24.2%)	124 (26.5%)	282 (25.5%)	0 (0.0%)	1527 (9.9%)
Survived to final FU, No. (%)	728 (75.8%)	344 (73.5%)	826 (74.5%)	47 (100.0%)	13900 (90.1%)
Among survivors to final FU[a]					
No final FU, No. (%)	75 (10.3%)	52 (15.1%)	84 (10.2%)	2 (4.3%)	437 (3.1%)
Final FU by GP, No. (%)	261 (35.9%)	147 (42.7%)	289 (35.0%)	1 (2.1%)	2052 (14.8%)
Final FU by patient / carer, No. (%)	393 (54.0%)	146 (42.4%)	455 (55.1%)	44 (93.6%)	11420 (82.2%)
Recorded at final FU[b]					
Referral to memory clinic or cognitive impairment as reason for no cognitive test, No. (%)	120 (18.4%)	67 (22.9%)	124 (16.7%)	0 (0.0%)	256 (1.9%)
Provided cognitive assessment, No. (%)	226 (34.6%)	65 (22.3%)	262 (35.3%)	31 (68.9%)	9009 (66.9%)
Age adjusted cognitive z score , mean (SE)	-0.64	-0.98	-0.61	-0.34	0.00

Abbreviations: FU, follow-up visit; SE, standard error.
[a]Percent is among survivors. [b]Percent is among survivors with final follow-up form.

Table S6. Numbers attempting and mean time taken for the individual components of the Healthy Minds cognitive assessment among the 2649 individuals included in the Healthy Minds analysis.

Component test	Number with complete test	Number with complete or partial test	Mean (SD) of time taken (minutes)[a]
Fluid intelligence	2548	2617	3.1 (0.9)
Trail making	2325	2483	3.1 (1.2)
Symbol digit substitution	2492	2528	3.1 (1.0)
Pair matching	2402	N/A[b]	2.9 (1.5)
Numerical memory	2437	N/A[b]	4.2 (1.6)

Abbreviations: SD, Standard deviation

[a] Time is the total time from the end of the previous test to the end of current test. Timings for individual tests greater than 15 minutes were considered outliers and were excluded from the mean (11 fluid intelligence tests, 17 trail making, 18 symbol digit substitution, 23 pairs matching and 19 numerical memory tests). [b] Partially completed tests were excluded for pair matching and numerical memory.

Table S7: Sensitivity analysis showing the observational association of the incidence of vascular and bleed events with dementia with different levels of adjustment for the predictors of dementia.

			Method of selection of additional adjustments[a]		
	Individuals with event and subsequently the broad dementia outcome	Individuals with the non-fatal event	Backwards P_{sel}=0.2[b]	Backwards $P_{sel=}$0.001	Stepwise P_{sel}=0.001
Non-fatal events			RR (95% CI)	RR (95% CI)	RR (95% CI)
Serious vascular events					
Non-haemorrhagic stroke[c]	62	362	2.76 (2.12-3.61)	2.86 (2.19-3.72)	2.89 (2.22-3.76)
TIA (and no stroke)	48	283	2.50 (1.85-3.36)	2.49 (1.85-3.35)	2.43 (1.81-3.27)
Myocardial infarction	30	374	1.54 (0.97-2.43)	1.55 (0.98-2.45)	1.53 (0.97-2.42)
Revascularization					
Coronary	32	528	0.87 (0.56-1.36)	0.88 (0.56-1.36)	0.88 (0.57-1.37)
Non-coronary	26	187	1.52 (1.01-2.28)	1.61 (1.08-2.40)	1.49 (1.00-2.23)
Major Bleed					
Intracranial bleed[c]	16	61	4.06 (2.43-6.77)	3.92 (2.37-6.49)	4.23 (2.56-7.00)
Gastointestinal bleed	27	218	2.12 (1.43-3.14)	2.20 (1.49-3.24)	2.17 (1.47-3.20)
Other major bleed	16	229	1.03 (0.63-1.71)	1.08 (0.66-1.78)	1.10 (0.67-1.81)
Serious vascular event	133	990	2.35 (1.92-2.87)	2.39 (1.96-2.92)	2.39 (1.96-2.92)
Revascularization	55	694	0.94 (0.70-1.27)	0.97 (0.72-1.30)	0.94 (0.70-1.26)
Major Bleed	59	496	1.89 (1.44-2.48)	1.96 (1.50-2.57)	1.99 (1.52-2.60)

[a] See Table S4 for details of forced and selected variables. [b] As in Figure 3. [c] Non-disabling events (analyses are censored at disabling stroke or intracranial bleed). Intracranial bleed includes haemorrhagic stroke.

Table S8: Estimated benefit for the broad dementia outcome of the effects of low dose aspirin on events in the ASCEND study

Non-fatal incident vascular and bleed events (censored at broad dementia outcome and disabling intracranial event)	Numbers with event (%)[a]		Percent avoiding event on aspirin	Rate ratio for broad dementia outcome (95% CI)	
	Aspirin (N=7714)	Placebo (N=7713)		Per event avoided[b]	In aspirin arm, through effect on the event
Serious vascular event	473 (6.1%)	517 (6.7%)	0.57%	0.43 (0.35-0.52)	0.995 (0.994-0.996)
Revascularization	326 (4.2%)	368 (4.8%)	0.55%	1.06 (0.79-1.43)	1.000 (0.999-1.002)
Major bleed	283 (3.7%)	213 (2.8%)	-0.91%	0.53 (0.40-0.69)	1.006 (1.003-1.008)
Net effect expected from events avoided or caused					1.001 (0.998-1.004)
Observed effect of aspirin in randomized comparison					0.910 (0.807– 1.025)

[a] Censoring at the broad dementia outcome or disabling intracranial event. [b]From inverse of rate ratios associated with having an event in Figure 3.

Figure S1. Association of Healthy Minds and TICSm+VF cognitive function z scores at end of study with the first subsequent recording of the broad dementia outcome.

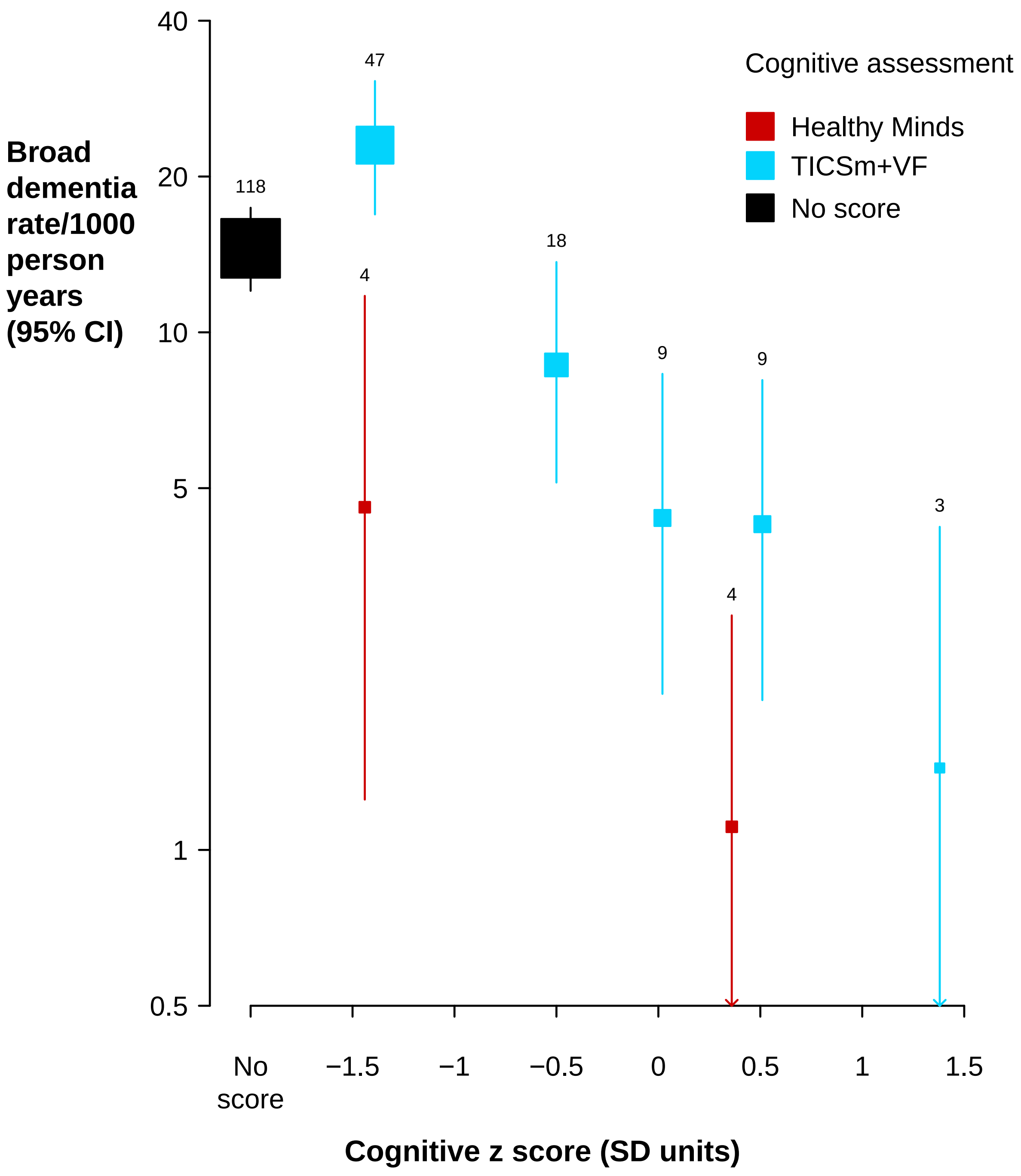

The TICSm+VF groups are fifths of the cognitive z score. The Healthy Mind groups are the lowest fifth of the cognitive z score and the remainder. Numbers above the points are the number of dementia cases in each group.

Figure S2. Observational association of the incidence of vascular and bleed events with dementia within strata of the number of hospital admissions for other causes during two−year age−at−risk intervals

Non−fatal incident event[a] and stratum by number of hospitalisations for other causes[b]	Participants with the broad dementia outcome / Person years: Following non−fatal event	Participants with the broad dementia outcome / Person years: Total	Rate ratio for the broad dementia outcome (95% CI)[c]
Serious vascular events			
No hospitalisations	47/1317	509/74595	2.61 (1.88, 3.62)
One hospitalisation	25/962	236/24254	2.07 (1.32, 3.24)
Two or more hospitalisations	61/1610	318/25901	2.47 (1.81, 3.38)
Adjusted for hospitalisations	133/3888	1063/124750	2.35 (1.92, 2.87)
Adjusted for alternate hospitalisation definition[d]	133/3888	1063/124750	2.34 (1.91, 2.86)
Revascularization			
No hospitalisations	20/993	509/74386	1.00 (0.61, 1.63)
One hospitalisation	7/717	233/24472	0.60 (0.27, 1.33)
Two or more hospitalisations	28/1195	321/25892	1.05 (0.68, 1.62)
Adjusted for hospitalisations	55/2905	1063/124750	0.94 (0.70, 1.27)
Adjusted for alternate hospitalisation definition[d]	55/2905	1063/124750	0.94 (0.69, 1.26)
Major bleeds			
No hospitalisations	16/664	507/74359	1.55 (0.92, 2.60)
One hospitalisation	13/422	237/24286	2.22 (1.22, 4.03)
Two or more hospitalisations	30/942	319/26105	2.16 (1.46, 3.21)
Adjusted for hospitalisations	59/2028	1063/124750	1.89 (1.44, 2.48)
Adjusted for alternate hospitalisation definition[d]	59/2028	1063/124750	1.87 (1.43, 2.45)

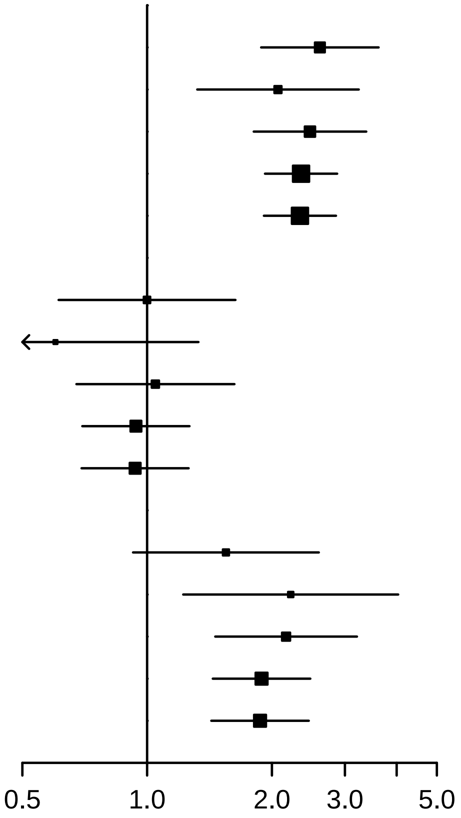

Abbreviations: CI, confidence interval;
[a] Non−disabling events (analyses are censored at disabling stroke or intracranial bleed).[b] Excludes admissions with a relevant dementia related diagnoses (Table S1) in the primary diagnostic position. [c] Poisson regression adjusted for two−year age−at−risk group, sex, randomized treatment, time exposed to other bleed and cardiovascular events of interest and baseline predictors of dementia, restricted to those with electronic hospital episode linkage. [d] Includes all hospital admissions.

Figure S3. Observational association of the incidence of vascular and bleed events with various dementia outcomes

	Total events [a]	Narrow dementia outcome (N=477): Events with subsequent outcome	Narrow dementia outcome (N=477): Rate ratio (95% CI) [b]	Broad dementia outcome (N=1063): Events with subsequent outcome	Broad dementia outcome (N=1063): Rate ratio (95% CI) [b]	Broad dementia outcome or care home (N=1190): Events with subsequent outcome	Broad dementia outcome or care home (N=1190): Rate ratio (95% CI) [b]
**Serious vascular events**							
Non−haemorrhagic stroke[c]	368	38	3.39 (2.39, 4.80)	62	2.76 (2.12, 3.61)	72	2.86 (2.24, 3.67)
TIA (and no stroke)	290	24	2.46 (1.61, 3.76)	48	2.50 (1.85, 3.36)	50	2.29 (1.71, 3.06)
Myocardial infarction	379	12	1.25 (0.61, 2.56)	30	1.54 (0.97, 2.43)	36	1.69 (1.11, 2.57)
**Revascularization**							
Coronary	538	14	1.05 (0.55, 2.03)	32	0.87 (0.56, 1.36)	36	0.83 (0.54, 1.26)
Non−coronary	192	12	1.39 (0.76, 2.53)	26	1.52 (1.01, 2.28)	30	1.53 (1.05, 2.24)
**Major bleeds**							
Intracranial bleed[c]	63	8	3.97 (1.92, 8.24)	16	4.06 (2.43, 6.77)	18	4.10 (2.53, 6.64)
Gastrointestinal bleed	224	11	1.70 (0.91, 3.17)	27	2.12 (1.43, 3.14)	31	2.09 (1.45, 3.02)
Other major bleed	233	7	1.07 (0.50, 2.29)	16	1.03 (0.63, 1.71)	18	1.01 (0.63, 1.62)
**Combined events**							
Serious vascular event	1010	71	2.63 (2.00, 3.47)	133	2.35 (1.92, 2.87)	150	2.33 (1.93, 2.82)
Revascularization	709	25	0.97 (0.63, 1.51)	55	0.94 (0.70, 1.27)	63	0.95 (0.72, 1.25)
Major bleed	508	26	1.77 (1.18, 2.67)	59	1.89 (1.44, 2.48)	67	1.87 (1.45, 2.41)

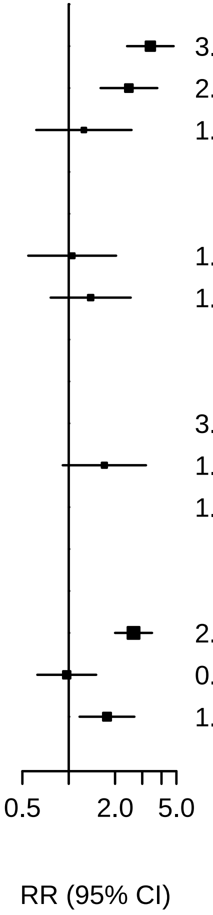

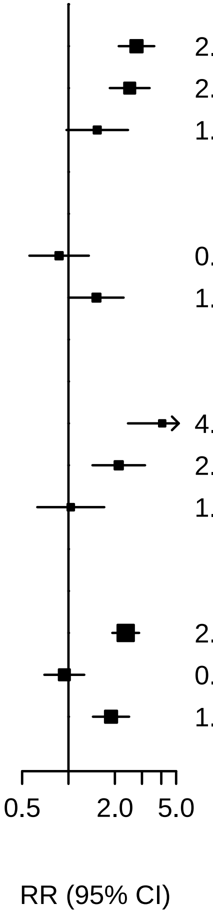

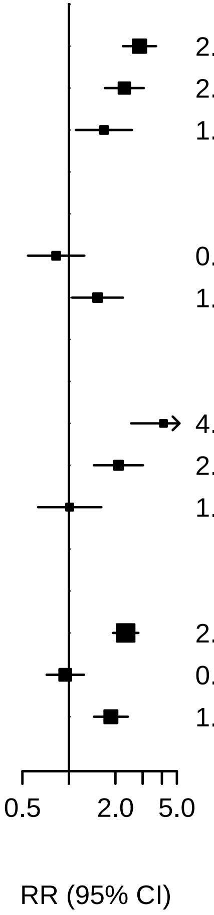

Abbreviations: RR, rate ratio; CI, confidence interval; TIA, transient ischaemic attack.
[a] Censored at the narrow dementia outcome, disabling stroke or intracranial bleed (differs from the total in Figure 3 which is censored at the broad dementia outcome).[b] Adjusted for age, sex, randomized allocation, number of hospitalisations, incidence of the other non−fatal incident events, prior disease, and baseline predictors of dementia selected by backwards selection with p_{sel}=0.2 (Table S4).[c] Non−disabling events; intracranial bleed includes haemorrhagic stroke.
